# Revolutionizing X-ray Imaging: A Leap toward Ultra-Low-Dose Detection with a Cascade-Engineered Approach

**DOI:** 10.1021/acscentsci.4c01296

**Published:** 2024-11-13

**Authors:** Xin Song, Xinyuan Zhang, Tengyue He, Jiayi Wang, Hongwei Zhu, Renqian Zhou, Taimoor Ahmad, Osman M. Bakr, Omar F. Mohammed

**Affiliations:** Center of Excellence for Renewable Energy and Storage Technologies, Division of Physical Science and Engineering, King Abdullah University of Science and Technology (KAUST), Thuwal 23955-6900, Kingdom of Saudi Arabia

## Abstract

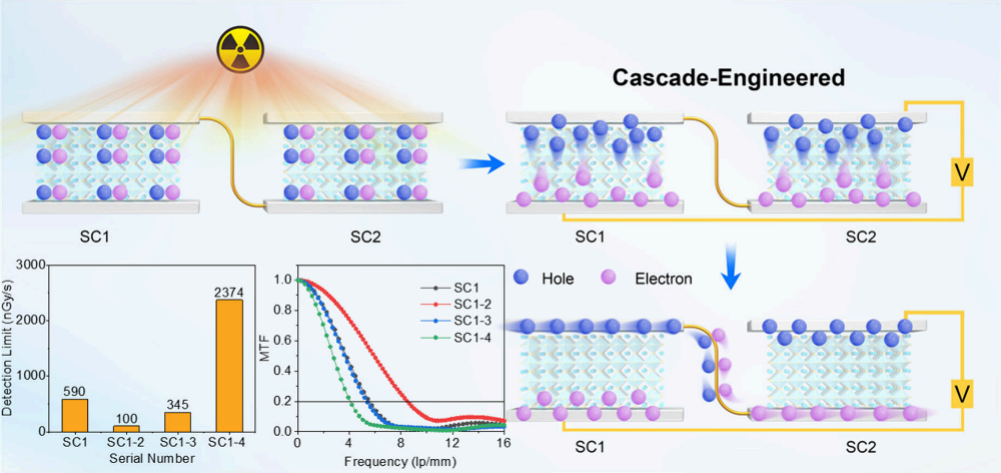

X-ray detection technology is essential in various fields, including medical imaging and security checks. However, exposure to large doses of X-rays poses considerable health risks. Therefore, it is crucial to reduce the radiation dosage without compromising detection efficiency. To address this concern, we propose an innovative cascade-engineered approach that uses two interconnected single-crystal devices to mitigate dark current and enhance the detection limit. Using laboratory-grown methylammonium lead bromide (MAPbBr_3_) perovskite single crystals, we engineered devices that significantly reduced detection thresholds and improved signal-to-noise ratios (SNRs). The detection threshold dropped from 590 nGy·s^–1^ with the conventional method to 100 nGy·s^–1^ using the cascade approach, surpassing the most recent record of 500 nGy·s^–1^ achieved for MAPbBr_3_ devices under nearly identical conditions. The dark current was halved compared to that of conventional devices, and spatial resolution improved from 5.6 to 8.5 lp·mm^–1^. Imaging trials confirmed improved resolution and effectiveness at low doses, highlighting the approach’s potential for medical diagnostics that prioritizes reducing radiation exposure without compromising image quality. The groundbreaking nature of this approach is highlighted by its adaptability across diverse electrical environments and crystal types, as evident in CdTe crystals, indicating its potential for widespread utilization in low-dose leakage monitoring and commercial X-ray devices.

## Abstract 2

This study explores a novel cascade-engineering device designed for low-dose X-ray detection.

## Introduction

X-rays, a fundamental form of electromagnetic radiation, have garnered widespread utility across diverse domains, including security screening, medical diagnostics, aerospace, and material analysis.^1−5^ The contribution of X-rays to society has been profound, particularly in the realm of medical imaging and X-ray leakage monitoring, where their utility is intimately connected to public healthcare. However, the inherent ionizing radiation and highly energetic nature of X-ray photons cause significant risks of irreversible harm to both biological entities and equipment. One of the major concerns is the potential for harm from exposure to high doses of X-rays. More specifically, prolonged or excessive exposure can cause a spectrum of adverse outcomes ranging from transient manifestations such as alopecia to more severe consequences, including carcinogenesis.^6,7^ Given the protracted time frame required for biological clearance of high radiation doses, the resulting damage may manifest as enduring and irreversible. In response to these concerns, concerted efforts within the scientific community have been directed toward attenuating the dosage of X-rays employed in medical imaging and analogous applications, including security checks. The overarching objective is to fulfill diagnostic imperatives while mitigating the associated risks to human health. Therefore, it is crucial to explore low-detection-limit X-ray monitoring devices and low-dose imaging. In recent years, great progress has been made in some efficient imaging design techniques. For instance, Su et al. explored lead-free, blue-emitting metal halides used in ceramic wafer scintillation screens, noted for their high luminous efficiency and potential as safer alternatives for high-performance X-ray imaging systems.^8^ Zhou et al. reported X-ray storage phosphors enhanced for flexible 3D imaging, detailing how defect manipulation and energy trap management significantly improve storage capacities and imaging resolution.^9^ Finally, Han et al. presented hybrid Eu(II)-bromide scintillators that utilize a 5d–4f band-gap transition, showcasing high light yields and low detection limits suitable for advanced X-ray imaging applications.^10^

These technological improvements are all about scintillator imaging, and research on direct X-ray detection technology remains at a bottleneck stage. Researchers have been examining various methods to lower the detection limits of X-ray detectors. One effective approach is to improve the quality of the crystal and/or surface passivation to modify the film and crystal.^11−14^ This approach reduces both ion migration and the concentration of internal defect traps, leading to a great enhancement in the sensitivity and a lowering of the detection limit. Recently, the Huang group^15^ reported a record-high mobility–lifetime product (μτ) of 1.2 × 10^–2^ cm^2^·V^–1^ of methylammonium lead bromide (MAPbBr_3_) perovskite single crystals. The lowest detectable X-ray dose rate was 0.5 μGy·s^–1^, with a sensitivity of 80 μC·Gy^–1^·cm^–2^. In this context, Liu et al.^16^ demonstrated a black α-phase formamidine lead iodide (FAPbI_3_) single crystal attained by lattice engineering via annealing in an ambient atmosphere. Their results exhibited a remarkably low detection limit of 1.1 nGy·s^–1^ and a high spatial resolution of 15.9 lp·mm^–1^. However, growing FAPbI_3_ single crystals proves to be challenging due to low experimental repeatability, and they can readily transition to a yellow δ-phase at room temperature, causing detector failure.^17^ An alternative effective strategy involved minimizing or suppressing the dark current to distinguish the signal current, thereby enhancing the distinction between the X-ray-induced current and inherent noise. Recent discoveries in high-resistance environmental organic macromolecules^18,19^ and bismuth-based perovskite materials^20,21^ have significantly improved the current stability and reduced the detection limits through their inherent high resistance. Nonetheless, despite these advancements, the detection limits of organic macromolecules have yet to surpass those of lead-based perovskite materials, primarily due to their less efficient charge transport properties.^22^ However, high-quality and stable bismuth-based perovskite single crystals also encounter issues such as challenges in the synthesis method, which is controllable and reliable, taking into account economic and environmental factors.^23^ Moreover, the implementation of 2D/3D heterojunctions provides a novel approach for suppressing electron–hole pair recombination in the dark at defect sites.^24,25^ In addition to these advancements, Lin et al.^26^ developed highly sensitive X-ray detectors utilizing an environmentally friendly, solution-grown thick BiI/BiI_3_/BiI (BixIy) van der Waals heterostructure. These devices showed an anisotropic X-ray detection response and achieved an impressively low detection limit of 34 nGy·s^–1^. However, this approach necessitates the application of interface engineering technology, which is challenging to control and replicate.^27^ Additionally, it requires lattice matching between various single crystals to ensure efficient charge transmission, limiting its universal applicability across numerous material systems.^28^

In this study, we introduce a novel and efficient strategy involving the cascade engineering of single-crystal devices aimed at reducing the dark current and improving the detection limit and spatial imaging resolution. We employed the MAPbBr_3_ perovskite and cadmium telluride (CdTe) single crystals to fabricate two distinct types of devices. This method successfully lowered the detection limit, thereby facilitating low-dose X-ray leakage monitoring. A key advantage of the cascade single-crystal device, with respect to a single-crystal device, is its ability to maintain the same signal current; this current is generated through the creation of electron–hole pairs upon X-ray irradiation. However, the cascade configuration is significantly lower and more stable in the dark, resulting in a substantially larger signal-to-noise ratio (SNR) and a reduced detection limit for the cascade device. Notably, in the case of the MAPbBr_3_ perovskite devices, we observed a decrease in the detection limit from 590 nGy·s^–1^ with the conventional method to 100 nGy·s^–1^ with the cascade approach. This single-pixel array detector exhibited exceptional spatial resolution, achieving a resolution of up to 8.5 lp·mm^–1^. Furthermore, we validated the effectiveness of this method under different bias voltages, thicknesses, and materials, demonstrating its adaptability in diverse electrical environments. These remarkable cascade-engineered methods could facilitate a new area for the incorporation of cost-effective, low-dose commercial detector arrays specifically designed for X-ray medical imaging.

## Results

**Figure 1:**
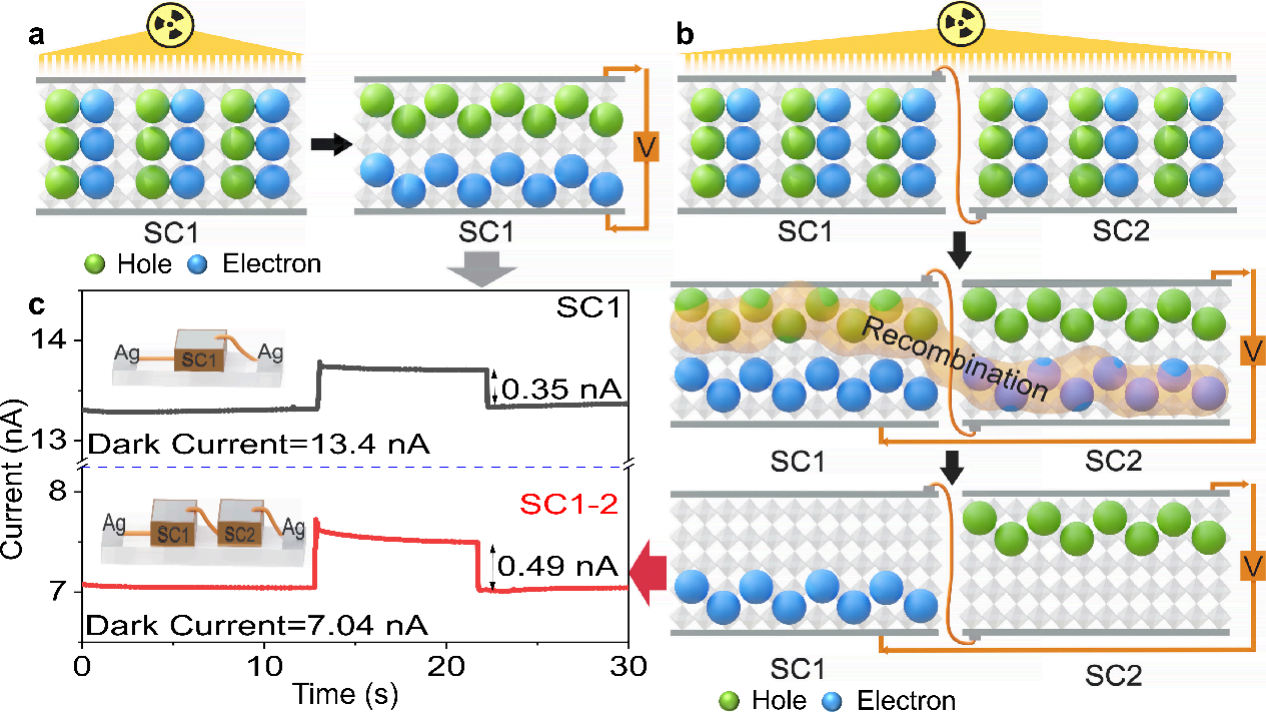
(a) Working principle diagram of a single-crystal photoconductive device under X-ray irradiation. (b) Working principle diagram of the cascade-connected two single-crystal photoconductive devices under X-ray irradiation. (c) Time-resolved photocurrent responses of the photoconductive MAPbBr_3_ SC1 and the cascade SC1–2 devices. To more clearly demonstrate the signal current generation process, the electron–hole pairs in parts a and b are the electron–hole pair concentrations obtained during irradiation, and the total electron–hole pair concentration of the crystals is not shown.

When X-rays are incident upon a semiconductor crystal device (Figure 1a), the photoelectric effect and Compton scattering are occured,^29^ which are proven to be crucial for solid-state X-ray detectors.^30,31^ These interactions generate free, high-energy electrons that create electron–hole pairs by ionizing atoms as they move through the semiconductor. These pairs are vital for X-ray detection, as they can be collected and converted into electrical signals (photocurrent). After the X-rays are stopped, the remaining current without photoinduced charge carriers is called the dark current. The difference between the photocurrent and dark current is the signal current, which reflects the net concentration of electron–hole pairs in the semiconductor, as shown in Figure 1a, and can be used to determine the device’s performance in response to X-ray photon excitation. When two nearly identical single crystals are connected in series and exposed to the same X-ray dose, they all generate equal net concentrations of electron–hole pairs, as depicted in Figure 1b. Applying the same direction of an external electric field across these crystals neutralizes the charge carriers at their junction, resulting in a net electron–hole pair count similar to that of a single crystal (Figure 1a). Thus, the signal current for cascade-connected crystals should match that of a single crystal. Additionally, this setup enhances electron and hole separation, improving the charge carrier concentrations. Despite this, the devices shown in Figure 1a and 1b exhibit different dark currents. According to the law of resistance,^32^ the resistance is directly proportional to the length, assuming that the resistivity and cross-sectional area remain constant. Therefore, the device in Figure 1b has a higher resistivity and will exhibit a lower dark current.

In the cascade-connected single-crystal devices, the working mechanism of these devices under X-ray irradiation can be summarized as follows: Cascade-connected single-crystal devices under X-ray irradiation generate electron–hole pairs, wherein the separation and diffusion of these pairs dictate the signal current. In series connections, charges are neutralized at the junction, segregating the electrons and holes into separate crystals but keeping the total pair generation constant in one single-crystal device. This device maintains signal current levels while significantly reducing the dark current, enhancing device detection performance by improving the signal-to-noise ratio (SNR).^21,33^ The current response tests of the MAPbBr_3_ single-crystal devices in Figure 1a (SC1) and 1b (SC1–2) are shown in Figure 1c. In this study, we utilized a cascade connection strategy involving multiple crystals to enhance the device performance. To provide a clear and concise description, we established a nomenclature based on the number of crystals connected in series. Specifically, devices with a single crystal are labeled SC1, SC2, SC3, and SC4. Devices consisting of two, three, and four crystals connected in series are designated as SC1–2, SC1–3, and SC1–4, respectively. This naming convention enables straightforward identification of the device configurations discussed throughout the article. The devices were subjected to X-ray irradiation at a dose rate of 33 μGy·s^–1^, with an applied electric field of 1 V·mm^–1^. The SC1 device generated a signal current of 0.35 nA, while the dark current was 13.4 nA. In contrast, for the cascade-connected device (SC1–2), the dark current was significantly reduced, nearly halving to 7.04 nA, while the signal current remained comparably stable at 0.49 nA. This result confirms the above theoretical analysis. Consequently, the cascade engineering of two single crystals can effectively reduce the dark current, thereby promoting charge separation and improving the SNR.

**Figure 2:**
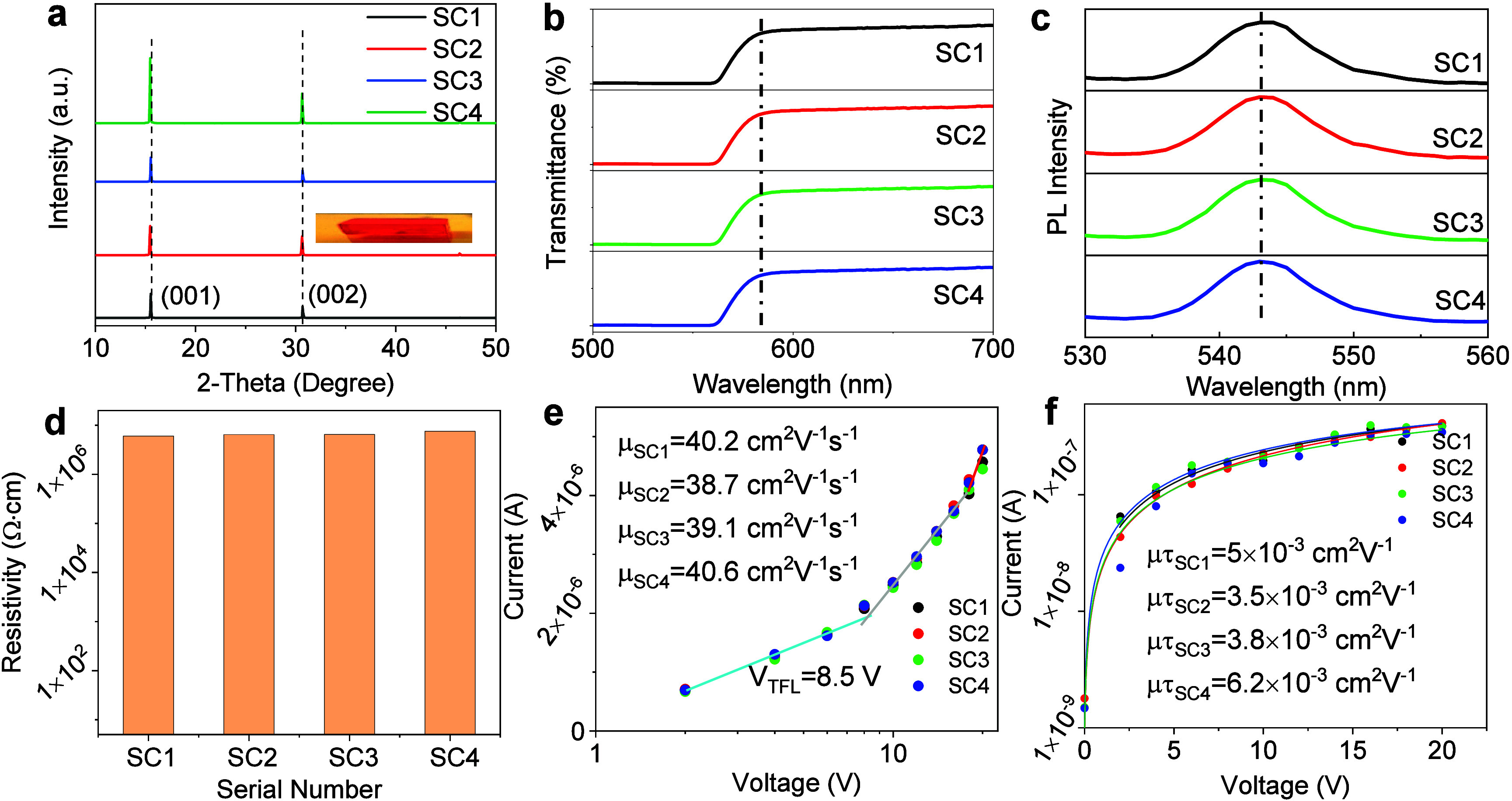
(a) XRD patterns of the four MAPbBr_3_ single crystals. (b) Transmittance spectra of the four 2-mm-thick MAPbBr_3_ single crystals. (c) PL spectra of four MAPbBr_3_ crystals at 510 nm excitation. (d) Resistivity results from the four MAPbBr_3_ single crystals. (e) Current–voltage curves of the four MAPbBr_3_ single devices using Space-charge-limited current (SCLC). (f) Bias-dependent photoconductivity of the four MAPbBr_3_ crystal devices. The Hecht equation was applied to fit the photocurrent conductivity data.

Initially, MAPbBr_3_ single crystals were grown by a temperature-controlled crystallization method, and the detailed procedures are outlined in the Supporting Information. Cracks in the crystal can greatly affect the device performance. Surface cracks create large defects that boost carrier recombination, lowering the efficiency. Cracks in the contact area may also cause short circuits, greatly impairing the device functionality. Ensuring uniformity in crystal quality and optoelectronic properties is a critical aspect of our experimental strategy. Four identical single crystals, each measuring 3 × 3 mm^2^ with a thickness of approximately 2 mm, were selected for the evaluation of fundamental photoelectric properties to confirm consistent performance across all samples. Note that these crystals exhibited a transparent, orange hue and a cubic phase.^34,35^ Surface characterization of the crystals was conducted using scanning electron microscopy (SEM), revealing smooth surfaces devoid of pinholes or wrinkles, as depicted in Figure S1. This high-quality surface morphology is advantageous for optimal interfacial contact in device applications. The crystal quality was further investigated using powder X-ray diffraction (PXRD). The PXRD patterns, shown in Figure 2a, closely aligned with previously reported literature references.^15,34,35^ Notably, all crystal faces of the four single crystals were indexed to the (00k) plane without any detectable impurity peaks, indicating uniform structural and packing characteristics. The narrow full-width at half-maximum (FWHM) values ranging from 0.06 to 0.08° for the PXRD peaks further confirmed the high crystal quality. These PXRD results corroborated the structural consistency across the four MAPbBr_3_ single crystals. The optical properties were assessed by using UV–visible spectroscopy, as presented in Figure 2b. The absorption spectra of all four MAPbBr_3_ single crystals displayed characteristic peak near the sharp drop in the band gap at approximately 580 nm; these results were consistent with the transmission data in Figure S2a. Moreover, the Tauc plots indicated a band gap of approximately 2.14 eV in Figure S2b, which is in good agreement with values reported in the literature.^35^ Photoluminescence (PL) measurements revealed an orange emission color with a peak intensity at approximately 543 nm and an FWHM of approximately 8 nm for all crystals upon 510 nm excitation, as shown in Figure 2c. These uniform absorption and PL characteristics across the four samples further demonstrate the consistency of the optical properties of the MAPbBr_3_ single crystals in our device engineering.

We further investigated the electronic properties of the four MAPbBr_3_ crystals. The detailed data are listed in Table S1. The average bulk resistivity of the four MAPbBr_3_ crystals was determined to be in the range of approximately 6.2 × 10^6^ to 6.5 × 10^6^ Ω·cm, as illustrated in Figure 2d. The carrier mobility (μ) and trap density (n_trap_) of the crystals were investigated by using the space-charge-limited current (SCLC) (Figure 2e). Remarkably, the n_trap_ values for all four crystals were found to be as low as approximately 6 × 10^9^ cm^–3^. These values were significantly lower, by approximately 4 orders of magnitude, compared to those of commonly used inorganic semiconductors, such as Si (n_trap_ = 10^13^–10^14^ cm^–3^),^36^ CdTe (n_trap_ = 10^11^–10^13^ cm^–3^),^37^ and CIGS (n_trap_ = 10^13^ cm^–3^).^38^ The mobility values for the four MAPbBr_3_ crystals were determined to be approximately 38.7 cm^2^ V^–1^ s^–1^; these values were comparable to that reported for MAPbBr_3_ perovskite single crystals in the literature.^35^ Additionally, we calculated the mobility–lifetime (μτ) products by fitting the intense light radiation conductivity data shown in Figure 2f using a modified Hecht equation.^39,40^ The μτ values for the four MAPbBr_3_ crystals are listed in Table S1. Among them, the lowest μt in SC2 was 3.5 × 10^–3^ cm^2^ V^–1^. This value approached those of state-of-the-art vapor-deposited cadmium zinc telluride (CdZnTe, 9.1 × 10^–3^ cm^2^ V^–1^).^41^ Overall, these results indicated the consistency of the electrical properties for the four MAPbBr_3_ single crystals. Then we implemented a vertical cascade-connected single-crystal strategy, as depicted in Figure S3, to explore the cascade crystals in series with an engineered approach involving two or more single crystals. The specific structure of the device is Ag/SCs/Ag. In addition, we used 100 μm copper wires to connect to the Ag electrodes during the series connection process to form a series circuit with four crystals. This design allows us to flexibly test single or multiple devices in series without having to prepare the devices again.

**Figure 3:**
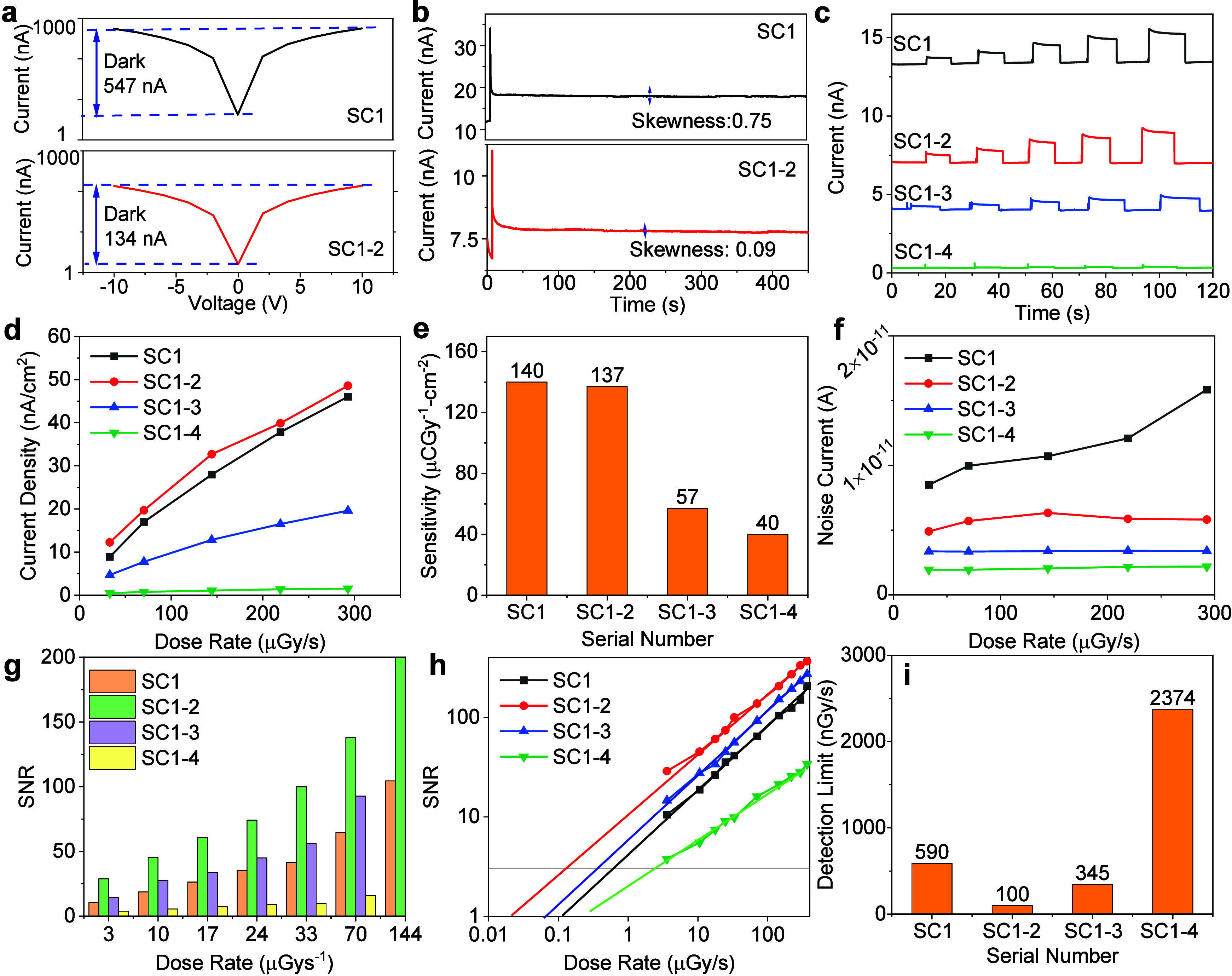
(a) Dark current of the cascade MAPbBr_3_ SC1 to SC1–4 devices. (b) Stability response of the MAPbBr_3_ SC1 and SC1–2 devices at a 2 V bias voltage under 480 μGy·s^–1^ for 450 s; other stabilities of the SC1–3 and SC1–4 devices are shown in Figure S4. (c) X-ray on–off response of the MAPbBr_3_ SC1 to SC1–4 devices at a 2 V bias voltage for 120 s. (d) Comparison of the signal currents of the MAPbBr_3_ SC1 to SC1–4 devices under a 2 V bias at doses ranging from 33 to 292 μGy·s^–1^. (e) Sensitivity comparison chart of the MAPbBr_3_ SC1 to SC1–4 devices. (f) Comparison of the noise currents of the MAPbBr_3_ SC1 and SC1–4 devices under a 2 V bias at doses ranging from 33 to 367 μGy·s^–1^. (g) SNRs of MAPbBr_3_ SC1 to SC1–4 at different doses (from 3 to 144 μGy·s^–1^). (h) Signal-to-noise ratio (SNR) of the MAPbBr_3_ SC1 to SC1–4 devices as a function of dose rate from 3 to 367 μGy·s^–1^. The detection limit was obtained by an epitaxy method. (i) Detection limit dose comparison chart of the MAPbBr_3_ SC1 to SC1–4 devices when the SNR is 3 under a 2 V bias.

Initially, we connected the four MAPbBr_3_ single crystals in series for the X-ray detectors. The dark-state current–voltage (I–V) curves for the four MAPbBr_3_ devices are depicted in Figure 3a. In this study, the dark current range of device SC1 demonstrated the highest value, reaching 547 nA. In contrast, devices SC1–2, SC1–3, and SC1–4 displayed a progressive decrease in dark current, with values of 134, 90, and 50 nA, respectively, providing clear experimental evidence for the decrease in the dark current due to the cascade-engineered strategy. These results highlight the reduction of the dark current by a cascade-engineered strategy possibly due to the increase in resistance. Next, we assessed the current stability of four distinct devices under continuous X-ray radiation for 450 s, as depicted in Figure 3b and Figure S4. Devices SC1–2 exhibited notable stability in their current response (the skewness value was 0.09), while the skewness values of SC1, SC1–3, and SC1–4 were 0.75, 0.45, and 0.76, respectively, which showed a gradual decline. It should be noted that the devices with two cascade-connected crystals demonstrated better stability, indicating an optimal balance between the connections and performance. Factors contributing to this decline included complications associated with the use of copper wires, an increase in the resistance, and limited charge transfer due to long cascade connections. Figure 3c shows the current–time (I–t) curve under 2 V bias at X-ray dose rates ranging from 33 to 367 μGy·s^–1^ for all devices. Notably, SC1–2 exhibited a dark current of 7 nA, approximately half that of SC1 (13 nA). Further reductions were observed in SC1–3 and SC1–4, with values of 4 and 3 nA, respectively. These results confirmed that connecting multiple single crystals in series reduced the dark current of the device, but the number of series connections also determined the stability of the device. The X-ray signal current density showed a linear increase with increasing X-ray dose rate under a 2 V bias (Figure 3d). Sensitivities of 140, 137, 57, and 40 μC·Gy^–1^·cm^2^ for SC1 to SC1–4, respectively, at a 2 V bias were achieved (Figure 3e). Notably, SC1 and SC1–2 exhibited comparable sensitivities, while a noticeable decrease was observed in SC1–3 and SC1–4. Figure 3f illustrates the noise current for the four devices, with SC1–2 demonstrating a significant reduction compared with SC1, enhancing the signal-to-noise ratio (SNR). At a dose rate of 3 μGy·s^–1^, SC1–2 maintained an SNR of close to 30, which was significantly greater than that of SC1 (10.5), as depicted in Figure 3g. Additionally, SC1–3 and SC1–4 exhibited SNRs of 15 and 3.8, respectively, highlighting the superior performance of SC1–2. Furthermore, the common epitaxy method was applied to calculate the detection limit in the dose range from 3 to 367 μGy·s^–1^ in Figure 3h.^16,42,43^Figure 3i shows the detection limit comparison results for the four devices. SC1–2 had the lowest detection limit (100 nGy·s^–1^); it was six times lower than that of SC1 (590 nGy·s^–1^), emphasizing the pivotal role of the cascade-connected SC1–2 configuration in reducing dark currents and significantly impacting the reduction in the detection limit, which is one of the key components for X-ray leakage monitoring and commercial imaging applications.

To evaluate the applicability of this method to varying bias voltage conditions, experiments were conducted by using a relatively low bias voltage of 0.5 V (Figures S5–S7). The current–time (I–t) curves are illustrated in Figures S5 and S6. Notably, the SC1 device exhibited instability in the dark current, indicating potential ion migration issues. However, this instability was significantly reduced in the SC1–2 device, indicating enhanced reliability under low-voltage operation. As the number of cascade connections increased to 3 and 4, noticeable decreases in the current response and the photocurrent were observed, which could be attributed to the excessive resistance inherent in devices with more serial connections and a long carrier diffusion distance. We describe the detailed possible reasons in Figure S8. The X-ray photocurrent density versus the X-ray dose rate at 0.5 V applied bias showed a linear increase (Figure S7a). SC1 and SC1–2 exhibited similar linearity, indicating consistent sensitivities, as shown in Figure S7b; however, a notable deviation was observed in SC1–3 and SC1–4, indicating a decreased sensitivity and linearity. Furthermore, the noise current decreased as the number of devices connected in series increased, aligning with previous observations. The detection limits for devices SC1 through SC1–4 were determined, with values recorded as 750, 528, 660, and 8974 nGy·s^–1^, respectively, in Figure S7d. SC1–2 demonstrate the lowest detection limit, indicating superior performance in detecting low-intensity signals, and its sensitivity (80 μC·Gy^–1^·cm^–2^) is close to that of SC1 (85 μC·Gy^–1^·cm^–2^), highlighting the versatility and potential of the cascade connection approach in enhancing the device performance under varying voltage conditions for diverse X-ray detection applications. The consistency of this trend across different applied bias voltages—both at the higher voltage (as discussed earlier) and now demonstrated at 0.5 V—indicates the robustness of the cascade-engineered strategy. This versatility underscores the potential of the cascade connection approach in enhancing device performance across a broad spectrum of operational voltages, making it a valuable strategy for diverse applications in X-ray detection where varying voltage conditions may be encountered. Additionally, we investigated the performance of cascade MAPbBr_3_ single crystal devices with varying thicknesses, as detailed in Figure S9. More specifically, Figure S9a shows the dark current measurements for devices with thicknesses of 1 and 1.5 mm, as well as a cascade device formed by serially connecting these two. The cascade device exhibited the lowest dark current, illustrating the effectiveness of this approach for devices of different thicknesses. As depicted in Figure S9b, the photocurrent responses varied with the crystal thickness. However, the cascade configuration achieved the highest signal-to-noise ratio (SNR), as shown in Figure S9c, confirming the utility of the cascade method across a range of thicknesses. To verify the applicability of this method to different materials, we conducted X-ray detection experiments by connecting commercial CdTe (110-facet) single crystals in series. The comprehensive details are provided in the Supporting Information (Figures S10–S13). Finally, we verified the versatility of this method for different materials.

**Figure 4:**
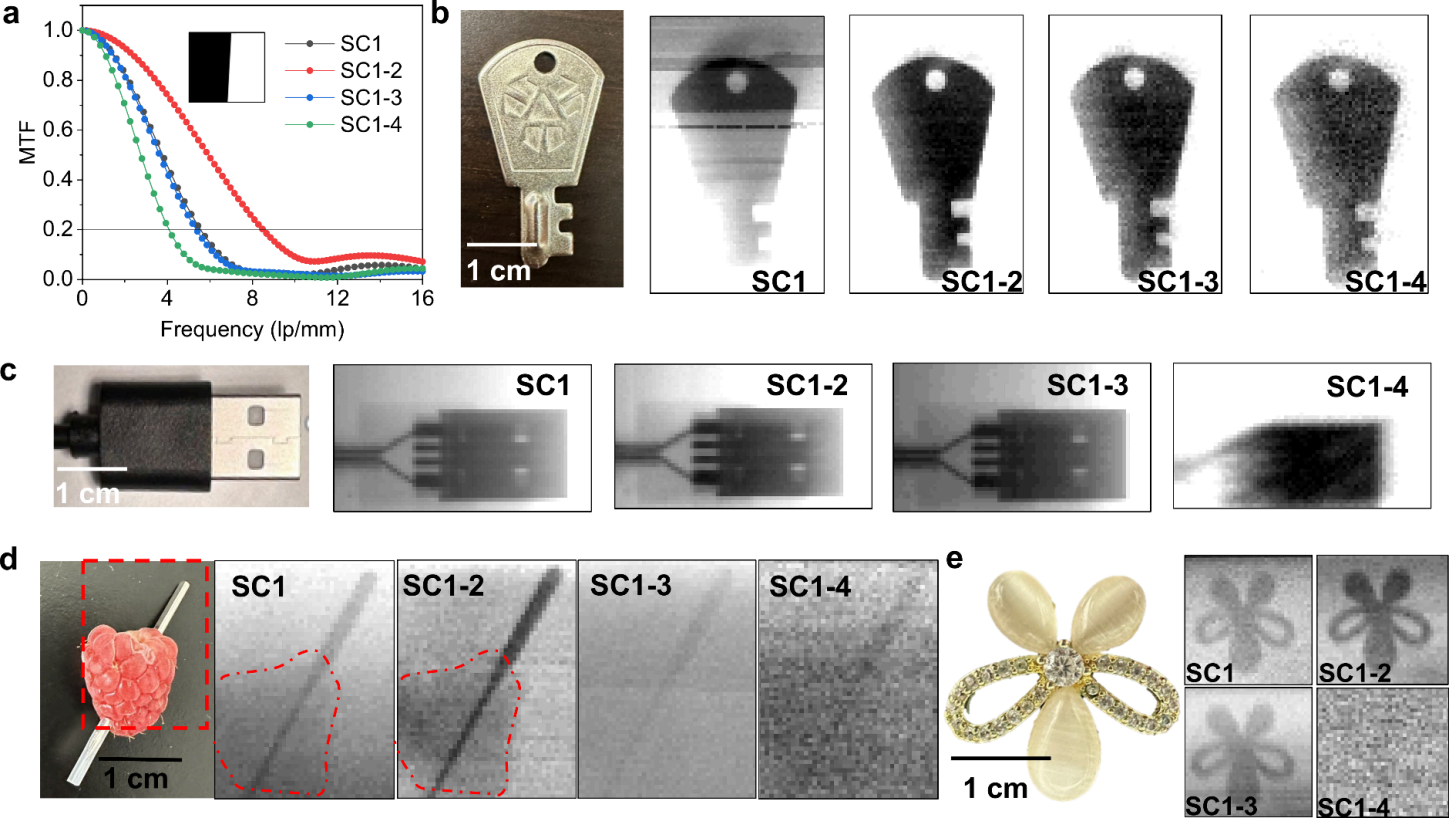
(a) Spatial frequency-dependent MTFs of the MAPbBr_3_ SC1 to SC1–4 devices. Inset: sloped edge image obtained from the SC1–2 device. (b) Actual and X-ray images of the key obtained by the MAPbBr_3_ SC1 to SC1–4 devices under 30 μGy·s^–1^. (c) Actual and X-ray images of the USB port of the MAPbBr_3_ SC1 to SC1–4 devices under 2.3 μGy·s^–1^. (d) Actual and X-ray images of a raspberry with a needle in MAPbBr_3_ SC1 to SC1–4 devices under a pressure of 1.9 μGy·s^–1^. (e) Actual and X-ray images of an earring by the MAPbBr_3_ SC1 to SC1–4 devices under 750 nGy·s^–1^.

The pursuit of low-dose detection is pivotal for enabling low-dose X-ray imaging under a 2 V bias voltage. An estimation of the imaging modulation transfer function (MTF) using the slanted-edge method revealed that SC1–2 demonstrated the highest spatial resolution (8.5 lp·mm^–1^) and was notably superior to SC1, SC1–3, and SC1–4, which were 5.6, 5.4, and 4 lp·mm^–1^, respectively, as illustrated in Figure 4a. The MTF test uses 50 × 50 μm^2^ as the pixel (Figure S14), and a detailed imaging device diagram is shown in the Supporting Information. Imaging experiments at various low doses were used to compare the performances of the four devices. Images captured at a dose rate of 3.1 μGy·s^–1^ using SC1 as presented in Figure 4b reveal the unclear “key” due to the pronounced current drift phenomenon (unstable current) affecting resolution under a high bias voltage and a high dose rate. In contrast, this issue of current drift is notably mitigated in devices SC1–2 to SC1–4, with SC1–2 producing a distinctly clearer image of the key than the others. As the number of cascade connections increased to 3 and 4, the imaging contrast decreased, indicating reduced differences between the photocurrent and dark current. Subsequent tests involved imaging a USB port at a dose of 2.3 μGy·s^–1^, as depicted in Figure 4c, where SC1–2 exhibited the highest imaging contrast and clarity and SC1–4 produced only a vague outline. The raspberries were imaged with a needle at 1.9 μGy·s^–1^, as shown in Figure 4d. SC1–2 could produce a more discernible image of the raspberry than SC1, which further highlighted the superior resolution and lower detection limit of SC1–2. At a dose of 750 nGy·s^–1^ (Figure 4e), SC1, SC1–2, and SC1–3 exhibited relatively clear contours, with the SC1–2 images appearing to be more distinct. The higher detection limit of SC1–4 resulted in vague imaging information. SC1–2 exhibits superior imaging contrast due to the enhanced cascade resistance, which significantly reduces dark current and noise. This configuration demonstrates not only improved current stability but also a lower noise current compared to SC1, thereby enhancing the resolution. These outcomes are in alignment with our initial theoretical predictions and highlight the potential of this method for low-dose, high-resolution imaging applications. Furthermore, we propose that the cascade connection approach is particularly beneficial for single crystals that inherently exhibit high dark currents and notable drift, such as 3D lead-based perovskites and 110-facet CdTe crystals. By effectively reducing both dark and noise currents, this method minimizes current drift, improves detection thresholds, and consequently enhances the spatial imaging resolution.

In this study, we effectively provided a mitigation strategy for controlling and tuning the dark current in X-ray detection by employing a cascade-engineered configuration. This method facilitated a notable reduction in the detection threshold of MAPbBr_3_ perovskite detectors from 590 to 100 nGy·s^–1^ with much better spatial imaging resolution, thereby highlighting its efficacy and versatility across diverse electrical contexts. In other words, a pivotal finding from our study was as follows: an optimal equilibrium between the sensitivity preservation and the achievement of the minimal detection threshold was elucidated and was associated with the preservation of the superior imaging quality; this was achieved through a controlled number of cascade interconnections and was notably capped at two single crystals. This groundbreaking discovery represents a significant shift in low-dose X-ray detection, with the potential to catalyze advancements in X-ray imaging technologies. Our research offers compelling evidence of the effectiveness of this approach in applications requiring minimal radiation exposure while ensuring excellent image quality. Therefore, our findings hold great importance for the field of low-dose leakage monitoring and foldable device detection as well as new methods for low-dose X-ray imaging.

## Supporting Information Available

Materials and methods; composition and structural characterization by SEM, XRD, and UV–vis; additional X-ray detection and imaging data; and an additional composition electrical performance table (PDF)
Transparent Peer Review report available (PDF)
