# Supplementary material for: Revolutionizing X-ray Imaging: A Leap toward Ultra-Low-Dose Detection with a Cascade-Engineered Approach
Source: ACS Cent Sci. 2024 Nov 13;10(11):2082–9. doi: 10.1021/acscentsci.4c01296 (PMC11613229; doi:10.1021/acscentsci.4c01296)
Supplement: Supplementary file 1 — oc4c01296_si_001.pdf [file oc4c01296_si_001.pdf]

Revolutionizing X-Ray Imaging: A Leap Toward Ultra-Low-Dose Detection with a Cascade-Engineered Approach

Xin Song, Xinyuan Zhang, Tengyue He, Jiayi Wang, Hongwei Zhu, Renqian Zhou, Taimoor Ahmad, Osman M. Bakr and Omar F. Mohammed*

Center of Excellence for Renewable Energy and Storage Technologies, Division of Physical Science and Engineering, King Abdullah University of Science and Technology (KAUST), Thuwal 23955-6900, Kingdom of Saudi Arabia

** Email: Omar.Abdelsaboor@kaust.edu.sa*

Materials and methods

Chemicals and reagents

$PbBr_2$ (99.999%) and anhydrous dimethylformamide were purchased from Sigma-Aldrich, and methylammonium bromine (MABr) was obtained from Luminescence Technology Corp, hydrobromic acid (HBr) (40%) were purchased from Aladdin Reagent. All the chemicals were used as received without further purification. CdTe single crystals were purchased from MTI Corporation (Materials Tech. Intl., USA).

Growth of the $MAPbBr_3$ single crystals

We used cooling and solution evaporation methods to prepare $MAPbBr_3$ single crystals:

Method 1. For the growth of $MAPbBr_3$ crystal, MABr and $PbBr_2$ with stoichiometric ratio of 1: 1 were dissolved in 20 ml HBr. The solution was maintained at temperature of 60 °C and stirred for 3 hours to ensure the completely dissolution of the solutes. The solution was then cooled to 30°C (3°C/day) for the crystal growth. After about 10-days growth, centimeter-sized $MAPbBr_3$ crystals could be obtained.

Method 2. The precursor MABr (0.748 g) was dissolved in anhydrous dimethylformamide (4 mL) in a 20 mL glass vial to form a clear solution. Then, $PbBr_2$ (2.452 g) was added into the glass vial with stirring to obtain a nearly saturated clear $MAPbBr_3$ solution. The glass vial was then placed onto a hotplate at 50 °C without disturbance for slow evaporation. Bulk $MAPbBr_3$ single crystals with dimensions in the centimeter range were obtained from the solution after 12 h.

X-ray diffraction (XRD)

The powder XRD analysis was performed on a Bruker D2 PHASER diffractometer equipped with a Cu Kα X-ray (λ=1.5406 Å) tube operated at 40 kV and 40 mA.

Scanning electronic microscopy (SEM)

The surface morphologies of the crystals were characterized by SEM (Quattro) at an acceleration voltage of 5 kV. The working distance is 10 mm.

Optical measurements

UV-Visible transmission spectra were acquired on a Pekin-Elmer Lambda 950 UV-Vis-NIR spectrophotometer operating in the 300 to - 800 nm region. Tauc plots were used to calculate the absorption from the transmission curve. Steady-state photoluminescence (PL) with an excitation wavelength of 510 nm were collected by a Horiba Fluromax-4 spectrofluorometer.

Space-charge-limited current (SCLC) analysis

The electron-only device configuration is Ag/$MAPbBr_3$/Ag with 80-nm-thick Ag. The electrode area is 4 mm^2, and the crystal thickness of $MAPbBr_3$ is 2 mm. Typically, a SCLC I-V curve shows two different regimes depending on the applied voltage, including a transition from the ohmic regime ($I{\sim}V^{n=1}$) at low voltages to the trap-filled regime ($I{\sim}V^{n>3}$) as the bias voltage increases and, eventually, to the quadratic Child's region ($I{\sim}V^{n=2}$). From the curve in the trap-filled regime ($I{\sim}V^{n>3}$), the trap density n_{trap} was calculated according to the following relation:(1)

$$n_{trap} = \frac{2V_{TFL}\varepsilon\varepsilon_0}{eL^2}$$

where V_{TFL} is the trap-filled limit voltage, L is the thickness of the crystal, ε_0 is the vacuum dielectric constant (8.854×10^{-14} F/cm), ε is the crystal dielectric constant and e is the electron charge (1.6×10^{-19} C).

From the Child's region ($I{\sim}V^{n=2}$) of the curve, the mobility was calculated according to the Mott-Gurney relation:(2)

$$J_D = \frac{9}{8}\varepsilon\varepsilon_0\mu\frac{V^2}{L^3}$$

where μ is the carrier mobility and V is the bias voltage.

Characterization of the trap photoconductivity

A photoconductivity analysis was carried out for the Au/ $MAPbBr_3$/Au device. A modified Hecht equation was used to fit the current-voltage curve, yielding a μτ product:(3)

$$I = \frac{I_0\mu\tau V}{L^2}\frac{1 - exp(-\frac{L^2}{\mu\tau V})}{1 + \frac{L}{V}\frac{S}{\mu}}$$

where I_0 is the saturated photocurrent, L is the thickness, V is the applied bias, and τ is the carrier lifetime.

Calculation of Signal-to-Noise Ratio

The determination of noise current involved calculating the photocurrent's standard deviation to derive the Signal-to-Noise Ratio (SNR). According to the IUPAC standard, the detection threshold is identified as the dose rate that generates a signal exceeding threefold the noise magnitude. Consequently, a dose rate corresponding to an SNR value of 3 was adopted as the detection limit under a specified electric field. The signal current calculation entailed deducting the mean dark current from the mean photocurrent. The noise current was ascertained by computing the photocurrent's standard deviation. Subsequently, the SNR was established using the specified equation.

$$I_{signal} = \bar{I}_P - \bar{I}_d$$

$$I_{noise} = \sqrt{\frac{1}{N}\sum_{i}^{N}(I_i - \bar{I}_p)^2}$$

$$SNR = I_{signal} / I_{noise}$$

X-ray detection

The X-ray source was purchased from Moxtek, the type is TUB00045-w01, tube voltage is 40kV. The mean energy is 29 kV. Interdigitated electrodes, 80 nm thick, composed of either Ag or Au, were deposited on the surfaces of $MAPbBr_3$ and CdTe single crystals using vacuum evaporation. These electrodes, covering an area of 2×2 mm^2, were used to fabricate vertical photodetectors, with non-conductive glass serving as the substrate. To construct the device, four $MAPbBr_3$ single crystals, each measuring 3×3×2 mm^3 and demonstrating similar performance, were interconnected using silver or gold paste. These connections were reinforced with copper wires, 100 μm in diameter and 1 cm in length, linking the lower surface of one crystal (SC1) to the upper surface of the subsequent crystal (SC2), as illustrated in **Figure S3**. Due to experimental limitations, the precise distance between each crystal was not controlled. However, to minimize resistance interference from the copper wires, their length was uniformly maintained at 1 cm across all connections. This careful management ensured that the total device area remained smaller than the X-ray spot size, which has a diameter of 2 cm. Following assembly, the device underwent a heat treatment at 110 °C on a hot stage for 30 minutes to solidify the silver or gold paste fully. The integrity of each connection was subsequently verified by measuring the resistance at each contact point using an Agilent U1272A multimeter, confirming the successful assembly of the device. The current in the electrode collection process is connected to the electrode through a 25 μm diameter probe. The X-ray-related measurements were recorded using a 2-mm-thick, Al-filtered, resulting in a dose range of 3.3 to 450 μGyair·s^{-1} by varying the magnitude of the tube current. An Accu-Gold DDX6-WL Sensor from Radcal was used to calibrate the X-ray dose rate. The X-ray responses of the devices were obtained using a Keysight B2902B semiconductor characterization system and a manual probe station under various bias voltages. All experiments were conducted in air without any encapsulation.

X ray imaging

A self-made X-ray imaging system composed of a shielded lead box, an X-ray source, an x-y step scanning system, and a precision source meter was used in this work (in **Figure S14**). We developed a custom Python software to control and record the system's operations. During the imaging process, the imaging object is manipulated using a two-dimensional moving stage, while the X-ray source and the device remain stationary. For instance, in the

imaging of raspberries, we initially calibrate the total movement range of the object to 20×30 mm^2, with a movement increment of 500 microns for each step. As the imaging object shifts, different sections intersect with the X-ray beam. Consequently, the X-rays absorbed by the device vary, thereby generating a corresponding current. By collecting the current data from each movement, a matrix ammeter is utilized to capture these variations. The differences in current values are then translated into an image, thus yielding the final imaging result. The size of the pixel can be determined by the step speed of moving the X and Y axes. In this article, the MTF test uses 50×50 um^2 as the pixel, so there are 400 pixels in one square millimeter. The pixel size in other image tests is 500×500 um^2.

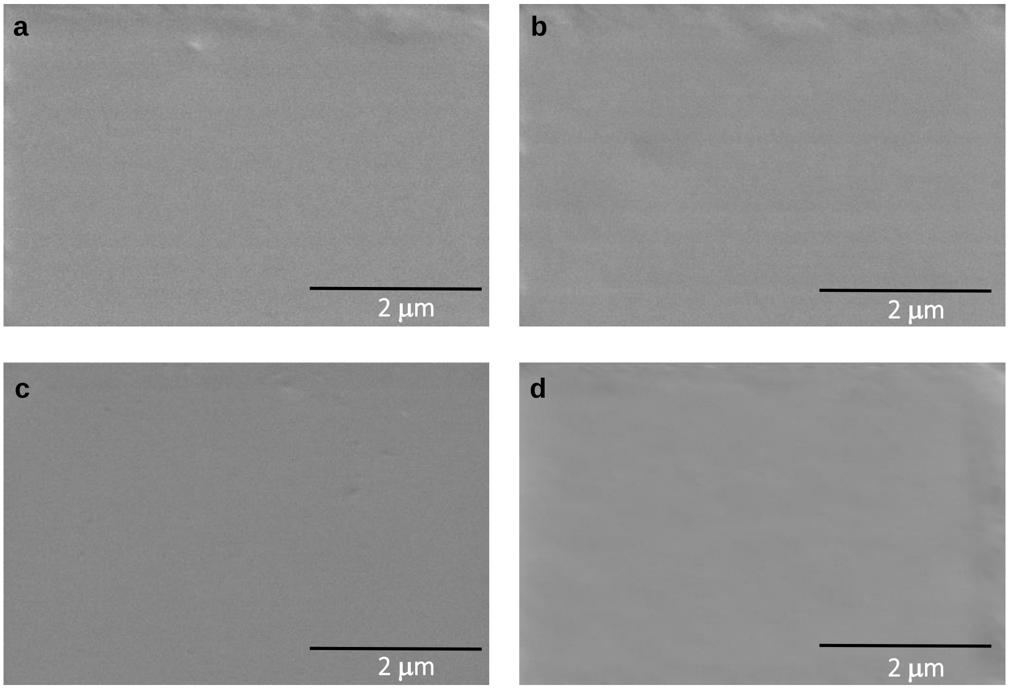

Figure S1. Scanning electronic microscopy (SEM) images of the surface of four $MAPbBr_3$ crystals under 2 μm magnifications.

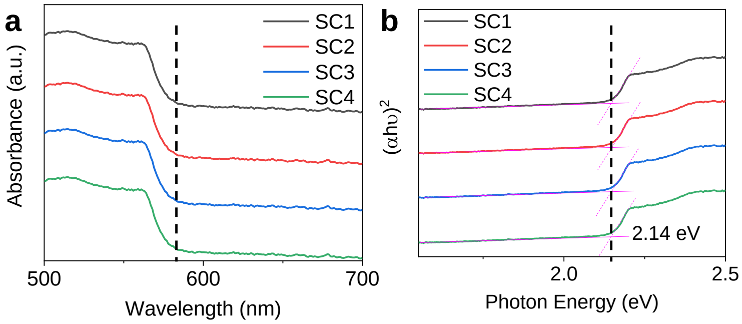

Figure S2. **(a)** The absorbance spectrum of the four 2 mm thick $MAPbBr_3$ single crystals. **(b)** Tauc plot of the four $MAPbBr_3$ single crystals.

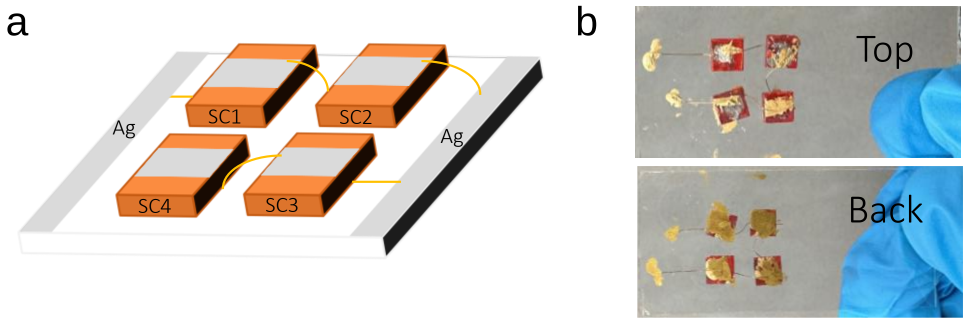

Figure S3. **(a)** Schematic illustration of the cascade-connected $MAPbBr_3$ and CdTe SC devices integrated on a glass substrate for X-ray response measurements. (b) The optical photo of cascade-connected $MAPbBr_3$ devices.

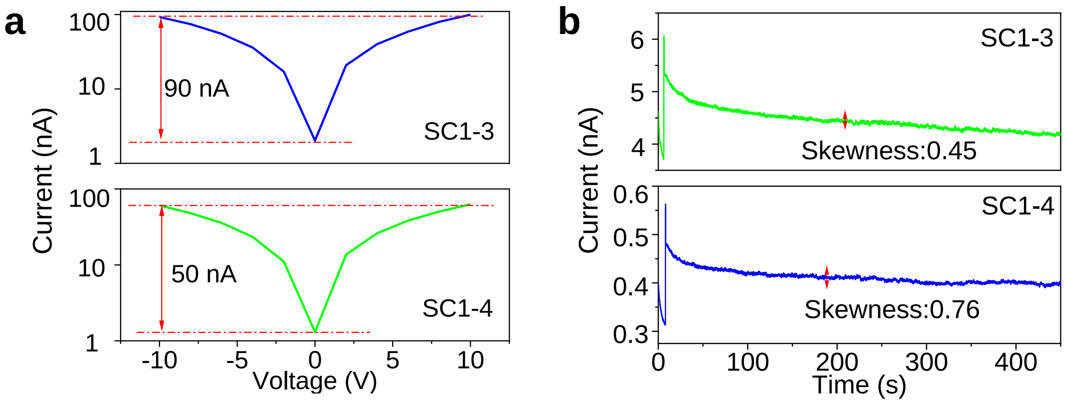

Figure S4. (a) Dark current of the $MAPbBr_3$ SC1-3 to SC1-4 devices. **(b)** Stability response of the $MAPbBr_3$ SC1-3 and SC1-4 devices at 2 V bias voltage under 480 μGy/s for 450 s.

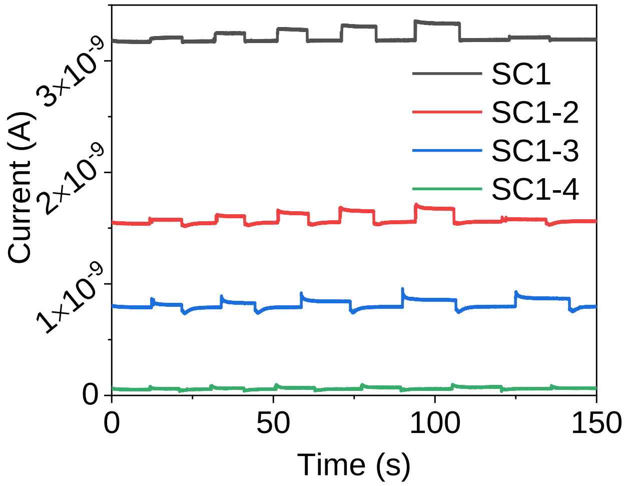

Figure S5. X-ray on-off response of the $MAPbBr_3$ SC1 to SC1-4 devices at 0.5 V bias.

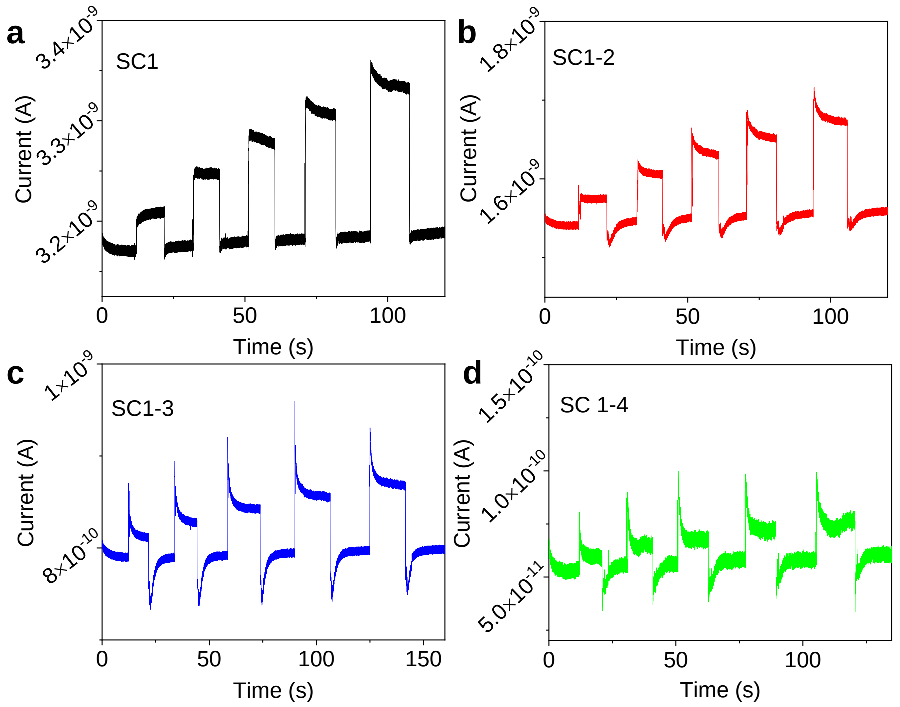

Figure S6. X-ray on-off response of **(a)** the $MAPbBr_3$ SC1, **(b)** SC1-2, **(c)** SC1-3, and **(d)** SC1-4 devices at 0.5 V bias.

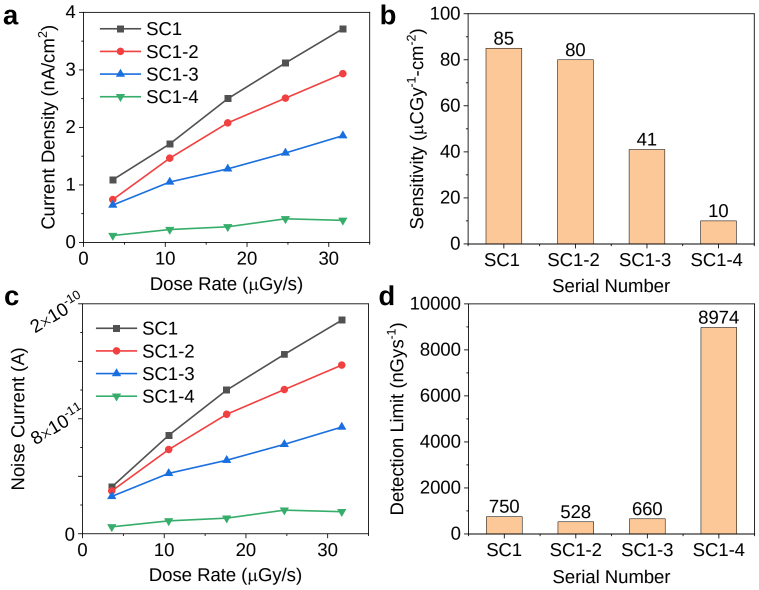

Figure S7. **(a)** Comparison of photocurrent of the $MAPbBr_3$ SC1 to SC1-4 devices under 0.5 V bias at the dose from 3.5 μGy/s to 32 μGy/s. **(b)** Sensitivity comparison chart of the $MAPbBr_3$ SC1 to SC1-4 devices under 0.5 V bias. **(c)** Comparison of noise current of the $MAPbBr_3$ SC1 to SC1-4 devices under 0.5 V bias at the dose from 3.5 μGy/s to 32 μGy/s. **(d)** Detection limit dose comparison chart of the $MAPbBr_3$ SC1 to SC1-4 devices when SNR is 3 under 0.5 V bias.

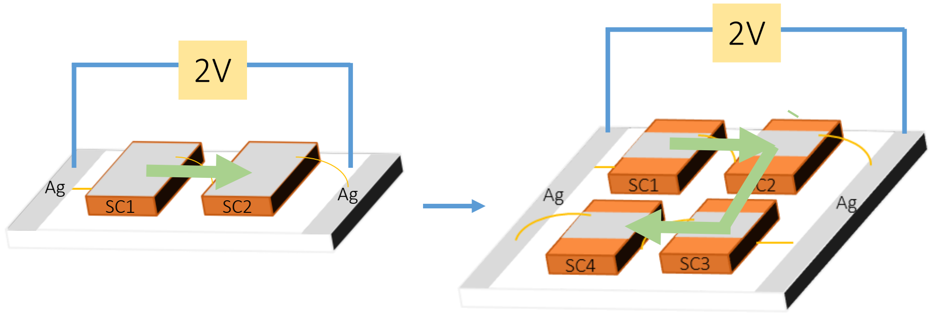

Figure S8. Charge transfer paths in two and four cascade devices. (Green line arrows are the path of neutralization from electrons and holes).

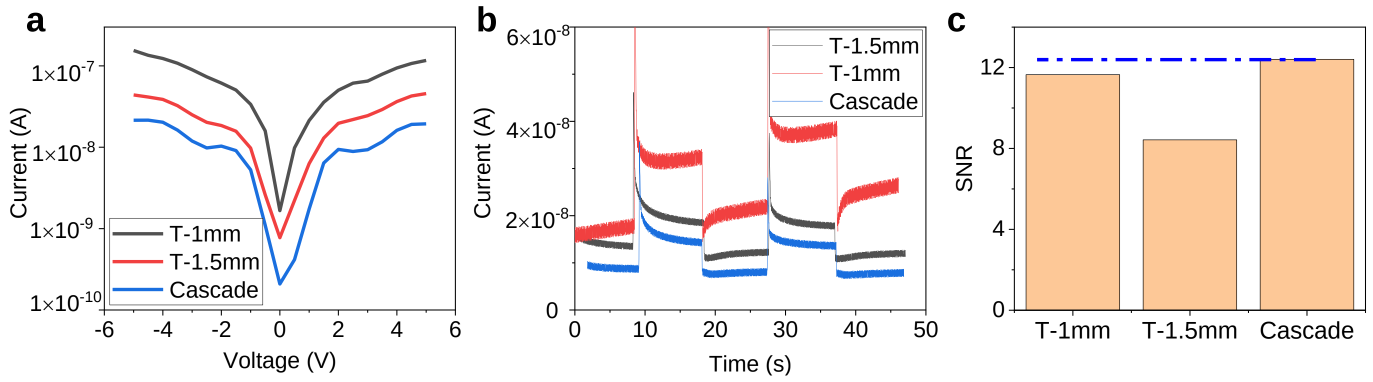

Figure S9. (a) Dark current of the $MAPbBr_3$ devices with 1mm, 1.5 mm and Cascade. **(b)** X-ray on-off response of the $MAPbBr_3$ with 1mm, 1.5 mm and Cascade devices at a 2 V bias under 2.8 $\mu Gy\cdot s^{-1}$ for 50 s. **(c)** SNRs of $MAPbBr_3$ at 2.8 $\mu Gy\cdot s^{-1}$.

In series connections of three or four crystals, we hypothesize that poor performance is due to excessive resistance. There are two possible reasons: (a) Increasing resistance to a certain extent reduces the device's dark current and improves the signal-to-noise ratio (SNR). However, excessive resistance impedes charge transfer, necessitating a balance between high resistance and high SNR. Experiments indicate that a low SNR is only achievable when two crystals are connected in series. (b) Furthermore, in series connections of multiple crystals, the internal offset charges are neutralized, and only the charges at the ends are transmitted (**Figure 1**). The relatively large distance between the crystals (SC1-4) negatively affects the transmission of charge due to the carrier diffusion length (**Figure S8** green line parts), leading to poor performance in more extended series connections.

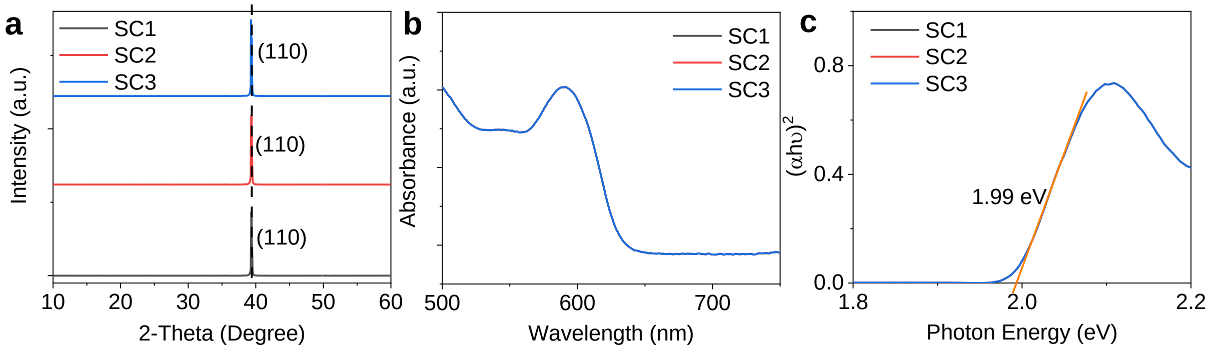

Figure S10. (a) XRD patterns of three CdTe single crystals. **(b)** The absorbance spectrum of the three 1 mm thick CdTe single crystals. **(c)** Tauc plot of three CdTe single crystals.

To verify the versatility of different materials of this cascade-engineered method, we use three commercial CdTe (110 face-oriented) single crystals with the same shape and the thickness of 1 mm to conduct the experiments. Powder X-ray diffraction (PXRD) patterns (**Figure S10a**) of the three crystals agree well each other and show the characteristic peaks of 110 face (39.4^o). The full-width at half-maximum (FWHM) for the 110 face is approximately 0.13^o - 0.14^o, which can ensure that each single crystal's crystallization quality is similar. The optical absorption properties (**Figure S10b**) and bandgap nature (**Figure S10c**) of three CdTe single crystals are evaluated. The absorption peaks of the three crystals are highly overlapping, and the absorption edges are all about 623 nm. The band gaps of the three crystals are 1.99 eV through the Tauc plot. This result is the same as previously reported.(4) Then three CdTe crystals with the same properties are connected in series to ensure that all single crystals can be irradiated by X-rays.

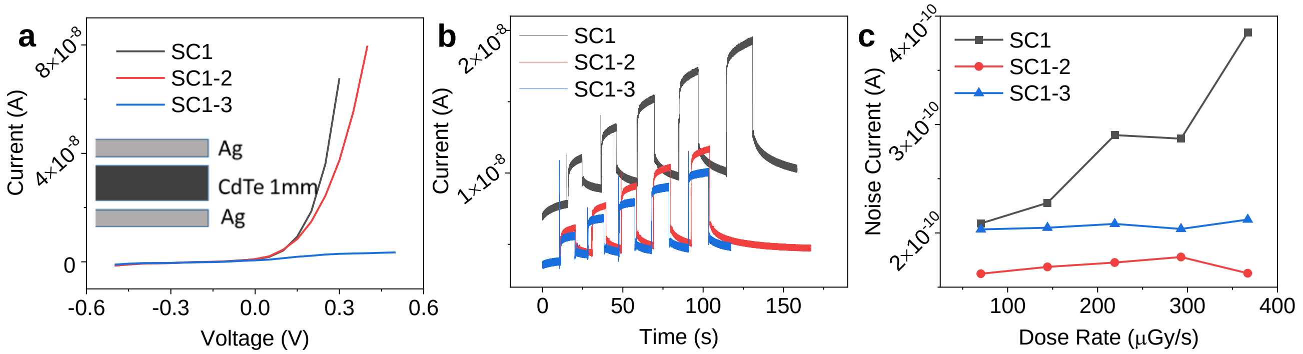

Figure S11. (a) Dark current of the CdTe SC1 to SC1-3 devices. **(b)** X-ray on-off response of the CdTe SC1 to SC1-3 devices at 2 V bias. **(c)** Comparison of noise current of the CdTe SC1 to SC1-3 devices under 2 V bias at the dose from 33 μGy/s to 367 μGy/s.

The device structure is shown in **FigureS3**. The current-voltage diagram of the three CdTe devices in the dark state as shown in **Figure S11a**. It can be clearly found that as the number of series single crystals increases, the dark current decreases. This phenomenon proved the feasibility of reducing dark current through series connection method, and subsequently we carried out X-ray detection experiments. **Figure S11b** showed the current-time I(t) curve under the 2V bias at the X-ray dose rate from 33 $\mu Gy \cdot s^{-1}$ to 367 $\mu Gy \cdot s^{-1}$ of three devices. The dark current of the CdTe SC1, SC1-2 and SC1-3 are as low as 7 nA, 3.3 nA and 3.2 nA at 2 V, respectively, the dark current of two single crystal device (CdTe SC1-2) is twice times higher than one single crystal (SC1) device, consistent with trends noted in $MAPbBr_3$ devices. Additionally, the dark current in CdTe SC1 gradually increased over time, suggesting that ion migration in the CdTe SC1 device leads to instability. In contrast, the dark current in CdTe SC1-2 became more stable as the resistance increases, which helps reduce noise current during X-ray detection. The dark currents of CdTe SC1-3 and SC1-2 are almost similar, but the photocurrent has decreased in CdTe SC1-3, indicating that the sensitivity and detection limit are lower than SC1-2. Furthermore, we calculate the noise current values for the three devices, which are shown in **Figure S11c**. The CdTe SC1-2 device exhibits minimal noise current (0.16 nA) that is twice as low as SC1 (0.25-0.38 nA), which demonstrated the series approach can achieve noise reduction. Ultralow dark current is key to improving the signal-to-noise ratio (SNR) of a device. Therefore, the CdTe SC1-2 devices are expected to be beneficial in terms of SNR and detection limit.

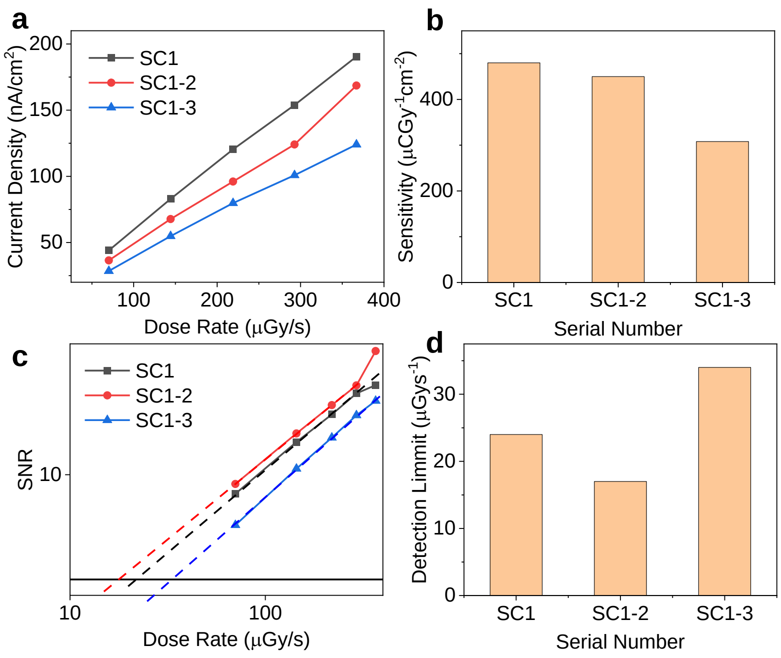

Figure S12. (a) Comparison of photocurrent of the CdTe SC1 to SC1-3 devices under 2 V bias at the dose from 33 μGy/s to 367 μGy/s. **(b)** Sensitivity comparison chart of t the CdTe SC1 to SC1-3 devices. **(c)** Signal-to-noise ratio (SNR) of the CdTe SC1 to SC1-3 devices as a function of dose rate. The detection limit was obtained by the epitaxy method. **(d)** Detection limit dose comparison chart of the CdTe SC1 to SC1-3 devices when SNR is 3 under 2 V bias.

We have plotted the photo-current difference against the dose from 33 μGy·s^{-1} to 367 μGy·s^{-1} under 2 V bias voltage in **Figure S12a**. The current gap of three devices increased linearly with the dose. The sensitivity can be roughly derived from the slope, which approached as 480, 450 and 308 μC·Gy^{-1}cm^{-2}, respectively. These observations demonstrate highly efficient charge extraction under X-ray excitation and the potential for low-cost single-crystal optoelectronics. The sensitivities of CdTe SC1-2 and SC1 are almost the same, but the sensitivity of CdTe SC1-3 has decreased then former devices, which is related to the decrease in photocurrent in **Figure S12b** above. This may be caused by the ultrahigh resistance of CdTe SC1-3. Signal-to-noise ratios (SNR) of the three devices are shown in **Figure S12c and S12d**. The detection limit values of CdTe SC1 to SC1-3, determined using the epitaxy method,(5) are 24, 17 and 34 μGy·s^{-1}. CdTe SC1-2 exhibits the lowest detection limit, corresponding to the previous low noise current. The detection limit of CdTe SC1-3 begins to increase, which is attributed to several reasons. First, the increase resistance leads to a decreasing conductivity. Another is the inability of the long charge transfer distance.

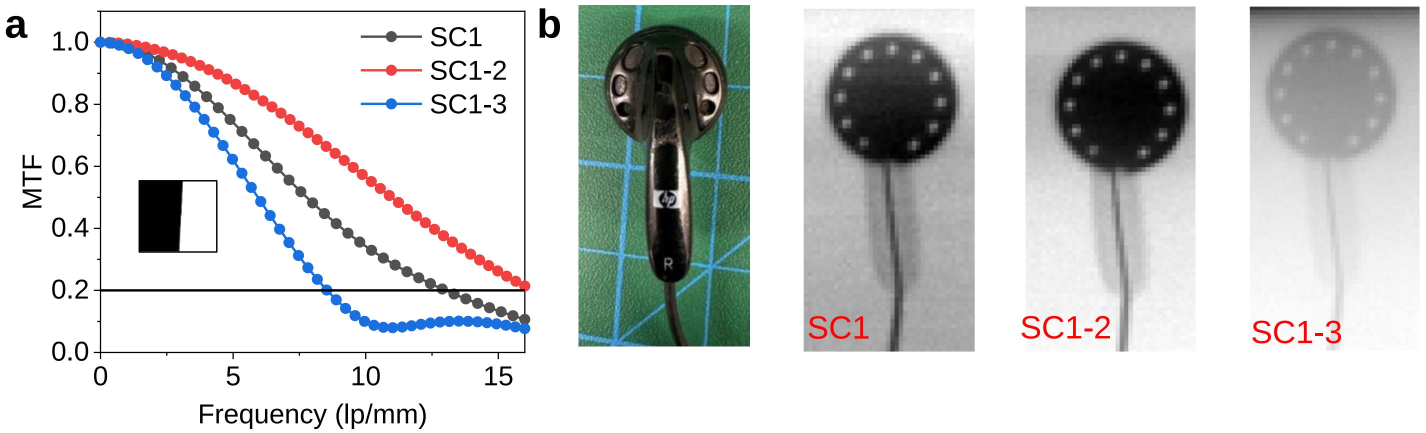

Figure S13. (a) Spatial frequency-dependent MTF of the CdTe SC1 to SC1-3 devices. Inset: sloped edge image obtained from the SC 1-2 device. **(b)** Actual and X-ray images of the headset by the CdTe SC1 to SC1-3 devices under 550 μGy/s.

For comparison, we have carried out the slanted edge method to calculate the spatial resolution of the three devices, which were 12, 16, and 8.3 lp·mm^{-1} from CdTe SC1 to SC1-3, respectively as shown in **Figure S13a**. Subsequently we also have imaged the headset using three devices, and the obtained images show clear organization inside with the dose rate of 550 μGy·s^{-1} (**Figure S13b**). The image of CdTe SC1 and SC1-2 show clear features, and the grayscale contrast of SC1-2 is more obvious and the imaging is clearer. The contrast of SC1-3 decreases and the image becomes worse. Finally, we proved that connecting two single crystals of CdTe in series can improve the detection limit without affecting the detection sensitivity that due to the reduction of noise current, which is beneficial to the detector working at lower doses and reducing the impact on biological and environmental pollution and damage.

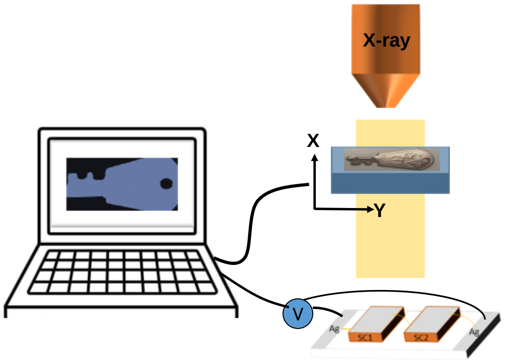

Figure S14. Single pixel imaging principle diagram.

Table S1. Electrical performances of four $MAPbBr_3$ single crystals.

	SC1	SC2	SC3	SC4
Resistivity (Ω cm)	6.2×10^{6}	6.4×10^{6}	6.5×10^{6}	6.4×10^{6}
V_{TFL} (V)	8.4	8.5	8.5	8.3
n_{trap} (cm^{-3})	6×10^{9}	6.07×10^{9}	6.07×10^{9}	6×10^{9}

μ ($cm^2V^{-1}s^{-1}$)	40.2	38.7	39.1	40.6
μτ (cm^2V^{-1})	5×10^{-3}	3.5×10^{-3}	3.8×10^{-3}	6.2×10^{-3}

Table S2. X-ray detection sensitivity and detection limit comparison of the series $MAPbBr_3$ SC devices with the reported perovskite devices under different electric fields.

Device type	Electric field ($V\cdot mm^{-1}$)	Energy (keV)	Sensitivity ($\mu C\cdot Gy_{air}^{-1}cm^{-2}$)	Detection limit ($nGy_{air}\ s^{-1}$)	Ref
Au/ $MAPbBr_3$ SC/C60/BCP/Au	0.1	50	80	500	(6)
Au/ $MAPbBr_3$ SC /Au	0.83		259.9		(7)
Au/ $MAPbBr_3$ SC/C60/BCP/Cr	6.1	120	3928.3	8800	(8)
Au/$MAPbBr_3$ SC/$CsPbBr_3$ SC heterojunction/Au	1250	120	201788	96	(9)
Au/BCP/C_{60} $MAPbBr_3$ SC /Si		50	21000	$<$100	(10)
AZO/$MAPbBr_3$/Au	50	80	529		(11)
Cu/BCP/C_{70} $MAPbBr_3$ Crystallites /NiO_x/ITO		60	488	2300	(12)
Ag/ $MAPbBr_3$ SC/Ag (This work)	1	40	137	100	This work

Table S3. X-ray detection sensitivity and detection limit comparison of the series $MAPbBr_3$ SC devices in this work.

Device type	Sensitivity ($\mu C\cdot Gy_{air}^{-1}cm^{-2}$)	Detection limit ($nGy_{air}\ s^{-1}$)
SC1	140	590
SC1-2	137	100
SC1-3	57	345
SC1-4	40	2374

Reference

1. R. H. Bube. Trap Density Determination by Space Charge-Limited Currents. *J. Appl. Phys.* **1962**, *33* (5), 1733-1737, DOI: 10.1063/1.1728818.
2. M. Kiy; P. Losio; I. Biaggio; M. Koehler; A. Tapponnier; P. Günter. Observation of the Mott–Gurney Law in Tris (8-hydroxyquinoline) Aluminum Films. *Appl. Phys. Lett.* **2002**, *80* (7), 1198-1200, DOI: 10.1063/1.1449527.
3. J. Androulakis; S. C. Peter; H. Li; C. D. Malliakas; J. A. Peters; Z. Liu; B. W. Wessels; J.-H. Song; H. Jin; A. J. Freeman; M. Kanatzidis. Dimensional Reduction: A Design Tool for New Radiation Detection Materials. *Adv. Mater.* **2011**, *23* (36), 4163-4167, DOI: 10.1002/adma.201102450.
4. L. Wang; R. Nughays; X. Song; T. Bian; M. N. Hedhili; J. Yin; O. M. Bakr; O. F. Mohammed. Crystallographic Orientation-Dependent Photo-Response of Planar Cadmium Telluride X-Ray Detectors. *Cell Rep. Phys. Sci.* **2023**, *4* (12), DOI: 10.1016/j.xcrp.2023.101723.
5. D. R. Shearer; M. Bopaiah. Dose Rate Limitations of Integrating Survey Meters for Diagnostic X-Ray Surveys. *Health Phys.* **2000**, *79*, S20-21, DOI: 10.1097/00004032-200008001-00007.
6. H. T. Wei; Y. J. Fang; P. Mulligan; W. Chuirazzi; H. H. Fang; C. C. Wang; B. R. Ecker; Y. L. Gao; M. A. Loi; L. Cao; J. S. Huang. Sensitive X-ray detectors Made of Methylammonium Lead Tribromide Perovskite Single Crystals. *Nat. Photonics* **2016**, *10* (5), 333-339, DOI: 10.1038/Nphoton.2016.41.
7. X. S. Geng; Q. X. Feng; R. Zhao; T. Hirtz; G. H. Dun; Z. Y. Yan; J. Ren; H. N. Zhang; R. R. Liang; H. Tian; D. Xie; Y. Yang; T. Ren. High-Quality Single Crystal Perovskite for Highly Sensitive X-Ray Detector. *Ieee Electr. Device L.* **2020**, *41* (2), 256-259, DOI: 10.1109/Led.2019.2960384.
8. J. Song; X. Feng; H. Li; W. Li; T. Lu; C. Guo; H. Zhang; H. Wei; B. Yang. Facile Strategy for Facet Competition Management to Improve the Performance of Perovskite Single-Crystal X-ray Detectors. *J. Phys. Chem. Lett.* **2020**, *11* (9), 3529-3535, DOI: 10.1021/acs.jpclett.0c00770.
9. F. Cui; P. Zhang; L. Zhang; Y. Hua; X. Sun; X. Li; G. Zhang; X. Tao. Liquid-Phase Epitaxial Growth of Large-Area $MAPbBr_3$–nCln/$CsPbBr_3$ Perovskite Single-Crystal Heterojunction for Enhancing Sensitivity and Stability of X-ray Detector. *Chem. Mater.* **2022**, *34* (21), 9601-9612, DOI: 10.1021/acs.chemmater.2c02266.
10. W. Wei; Y. Zhang; Q. Xu; H. T. Wei; Y. J. Fang; Q. Wang; Y. H. Deng; T. Li; A. Gruverman; L. Cao; J. S. Huang. Monolithic Integration of Hybrid Perovskite Single Crystals with Heterogenous Substrate for Highly Sensitive X-Ray Imaging. *Nat. Photonics* **2017**, *11* (5), 315-321, DOI: 10.1038/Nphoton.2017.43.
11. L. Li; X. Liu; H. Zhang; B. Zhang; W. Jie; P. J. Sellin; C. Hu; G. Zeng; Y. Xu. Enhanced X-ray Sensitivity of $MAPbBr_3$ Detector by Tailoring the Interface-States Density. *ACS Appl. Mater. Interfaces* **2019**, *11* (7), 7522-7528, DOI: 10.1021/acsami.8b18598.
12. J. Peng; K. Ye; Y. Xu; L. Cui; R. Li; H. Peng; Q. Lin. X-Ray Detection Based on Crushed Perovskite Crystal/Polymer Composites. *Sensor Actuat. A-Phys.* **2020**, *312*, DOI: 10.1016/j.sna.2020.112132.
