# Supplementary material for: Revolutionizing X-ray Imaging: A Leap toward Ultra-Low-Dose Detection with a Cascade-Engineered Approach
Source: ACS Cent Sci. 2024 Nov 13;10(11):2082–9. doi: 10.1021/acscentsci.4c01296 (PMC11613229; doi:10.1021/acscentsci.4c01296)
Supplement: Supplementary file 2 — oc4c01296_si_002.pdf [file oc4c01296_si_002.pdf]

oc-2024-01296f.R1

Name: Peer Review Information for "Revolutionizing X-Ray Imaging: A Leap Toward Ultra-Low Dose Detection with a Cascade-Engineered Approach"

First Round of Reviewer Comments

Reviewer: 1

Comments to the Author

In this work, the authors demonstrate an interesting detector configuration, by employing two interconnected single-crystal devices to mitigate the dark current and significantly enhance the charge separation. As a result, the device yielded substantial reductions in detection thresholds and improvement in SNRs. The results are convincing, which inspires future R&D of perovskite X-ray imaging/detection devices. I recommend its publication. A few comments are listed below.

1. It is suggested to investigate the combination of single crystals with different thickness.

2. If possible, the contact at electrode/perovskite can be further optimized.

3. Please elucidate mechanisms: why SC1-2 show the best imaging contrast in Figure 4, which has the smallest difference between the photocurrent and dark current.

Reviewer: 2

Comments to the Author

In this work, an innovative cascade-engineered approach was reported by utilizing the star materials of $MAPbBr_3$ perovskite crystals. The results demonstrated a great reduction in the dark current and X-ray detection thresholds along with enhanced spatial imaging resolution compared to traditional devices. Furthermore, detection limits were largely reduced from 590 to 100 $nGy \cdot s^{-1}$. This manuscript is interesting and innovative, and can be published on ACS Central Science after minor revisions.

The issues are listed below:

1. The identifications and definitions of SC1, SC2, SC3, and SC4 are not clear enough. Does SC3 refer to three cascade-engineered crystals? A clear definition is good for the reading and understanding when it first appeared.

2. Does this approach also useful for other single-crystals (MAPbI3, MA3Bi2I9), since the author mentioned that this innovative approach has proven to be adaptable to various crystal types? Some comments are expected for the future studies.

3. Will the crystal crack affect the property in cascade-engineered approach?

4. The format of y axis in Figure 3a and Figure 3b is different from Figure 3c. Why does author separate the data in Figures 3a,b while merged data in Figure 3c?

5. Some recent reports on the X-ray detection and imaging can be given a short description to compare the present technology, and some references are shown herein, Light: Science & Applications, 2024, 13, 222.; Advanced Materials, 2023, 35, 2212022.; Advanced Functional Materials, 2023, 33, 5, 2210735.

6. Reference format should be re-checked; for instance, Adv. Func. Mater. should be Adv. Funct. Mater..

Reviewer: 3

Comments to the Author

he results reported in this manuscript were interesting, but the novety of this mansucript was not well described.

Author's Response to Peer Review Comments:

KING ABDULLAH UNIVERSITY OF SCIENCE AND TECHNOLOGY
Division of Physical Sciences and Engineering, Thuwal 23955-6900, KSA

Omar F. Mohammed, *PhD, FRSC, FIMMM, IOP*
Associate Editor for ACS Applied Materials & Interfaces
Professor of Chemistry and Material Sciences and Engineering
Advanced Membranes & Porous Materials & KAUST Catalysis Centers
Division of Physical Sciences and Engineering
Room 3277, Level 3, Building 5
KAUST, Thuwal 23955-6900, KSA
Phone: +966 2 808 4491
E-mail: omar.abdelsaboor@kaust.edu.sa

Sept 29th, 2024

ACS Central Science
Deputy Editor

Dear Editor,

Thank you for your email on Sept 20th, 2024, regarding our manuscript oc-2024-01296f. We thank you so much for your attention to this work. We are also grateful to the two referees for their time and effort spent on this submission and for providing insightful comments and suggestions, which we have fully addressed in the revised version of the manuscript. Finally, as you will see in the subsequent paragraphs, we present our response to the reviewers' comments in a point-by-point manner.

I hope that you will find this manuscript significantly modified and improved, addressing all the reviewers' concerns and suitable for rapid and timely publication in *ACS Central Science*.

Sincerely,

Omar F. Mohammed, PhD, FRSC, FIMMM, IOP

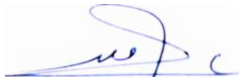

2019 - 2023 highly cited researcher (Web of Science)
Associate Editor for ACS Applied Materials & Interfaces

Point-by-point response to the editor's and reviewers' comments

Reponse to Editor

Q1. How were the contacts made to the crystals, and the interconnects positioned? Photographs of the device would substantially enhance the description.

Our reply: The contact points were established by making contact between the silver or gold electrodes and the surface of the single crystal. Interconnections were formed by connecting the silver electrodes using 100 μm copper wires. To clarify the device preparation process, we have included a photo of the cascade-connection $MAPbBr_3$ device in Figure S3b (see page S5) in the revised supplementary information.

Q2. What was the size of the device, and how far apart were the crystals positioned.

Our reply: In this study, we utilized four 3×3×2 mm^2 $MAPbBr_3$ single crystals connected in series. While we did not specifically control the distance between each crystal, we arranged them as closely as possible to minimize resistance from the length of the copper wires. Each connecting copper wire measured 1 cm in length, ensuring that the total area of the final device was smaller than the X-ray spot size (2 cm in diameter) to guarantee that the X-ray could fully irradiate all the crystals. Additional details are provided in the revised supplementary information in the X-ray detection section (see page S3).

Q3. The imaging layout is described only briefly, yet it plays an important role in the results. This needs to be described better in the revision.

Our reply: As suggested, we have provided a more detailed description of the imaging equipment layout and imaging experiments in the revised manuscript. Please refer to Figure S14 and the X-ray imaging section of the supplementary information (see pages S3-4).

Q4. The nomenclature used in the paper is not clear and needs to be better defined. SC1, SC2, etc.

Our reply: As suggested, we have included the abbreviations for "cascade crystals" and "single crystals" on pages 3-4 of the revised version of the manuscript.

Reponse to Reviewer 1

Comments

In this work, the authors demonstrate an interesting detector configuration, by employing two interconnected single-crystal devices to mitigate the dark current and significantly enhance the charge separation. As a result, the device yielded substantial reductions in detection thresholds and improvement in SNRs. The results are convincing, which inspires future R&D of perovskite X-ray imaging/detection devices. I recommend its publication. A few comments are listed below.

Our reply: We thank the reviewer for appreciating the importance of our work and recommending publication in *ACS Central Science* after minor revision. Reviewer#1 has raised a several minor concerns of clarifications, which we have fully addressed in both the revised versions of the MS and SI. Here are our responses to the reviewer's comments in a point-by-point fashion.

Q1. It is suggested to investigate the combination of single crystals with different thickness.

Our reply: As suggested, we conducted signal-to-noise ratio (SNR) tests on two single crystals of differing thicknesses connected in series and observed results consistent with those obtained from crystals of uniform thickness. We have included a detailed description of the experiments involving devices with varying thicknesses in series on page 7 of the revised manuscript and have supplemented Figure S9 in the revised supplementary information (see page S7).

Q2. If possible, the contact at electrode/perovskite can be further optimized.

Our reply: We thank the reviewer for the remark. We optimized the performance of the single crystal device by evaporating gold or silver onto the surface of the single crystal to precisely control the size,

thickness, and contact area of the electrode. Prior to conducting full tests, we utilized a resistance meter to preliminarily verify the quality of the contact. Subsequent testing of the dark current IV curve confirmed the presence of good ohmic contact between the electrode and the single crystal, as illustrated in Figure 3a (see page 8) and Figure S4 of the revised supplementary information on page S5. These details have been comprehensively documented now in the X-ray detection section of the revised supplementary information on page S3.

Q3. Please elucidate mechanisms: why SC1-2 show the best imaging contrast in Figure 4, which has the smallest difference between the photocurrent and dark current.

Our reply: We appreciate the reviewer for bringing this issue to our attention. The enhanced performance of SC1-2 can be linked to the increased cascade resistance, resulting in reduced current drift and improved current stability, along with lower noise current. We have elaborated on these explanations in the revised manuscript to better clarify the underlying mechanisms (refer to page 9).

Reponse to Reviewer 2

Comments

In this work, an innovative cascade-engineered approach was reported by utilizing the star materials of $MAPbBr_3$ perovskite crystals. The results demonstrated a great reduction in the dark current and X-ray detection thresholds along with enhanced spatial imaging resolution compared to traditional devices. Furthermore, detection limits were largely reduced from 590 to 100 $nGy \cdot s^{-1}$. This manuscript is interesting and innovative, and can be published on *ACS Central Science* after minor revisions.

Our reply: We appreciate the reviewer's recognition of the suitability of our findings for publication in *ACS Central Science* after minor revisions. Reviewer#2 made some constructive comments, which greatly improved the presentation of our work. Here is our response to the comments of the reviewer in a point-by-point fashion.

Q1. The identifications and definitions of SC1, SC2, SC3, and SC4 are not clear enough. Does SC3 refer to three cascade-engineered crystals? A clear definition is good for the reading and understanding when it first appeared.

Our reply: We thank the reviewer for the remark. As suggested, we have included the abbreviations for "cascade crystals" and "single crystals" on pages 3-4 of the revised manuscript.

Q2. Does this approach also useful for other single-crystals ($MAPbI_3$, $MA_3Bi_2I_9$), since the author mentioned that this innovative approach has proven to be adaptable to various crystal types? Some comments are expected for the future studies.

Our reply: We thank the reviewer for this suggestion. Connecting $MAPbI_3$ or $MA_3Bi_2I_9$ single crystals in series is indeed feasible and may yield satisfactory performance. However, it is important to highlight the existing issues and limitations associated with each crystal that must be addressed before considering a cascade design. For instance, the reproducibility of $MAPbI_3$ single crystals is currently low, and their phase stability is inadequate, which restricts their commercial application. Additionally, $MA_3Bi_2I_9$ single crystals exhibit inherently poor charge transport capabilities, making a cascade connection unnecessary. In the conclusion of the manuscript, we have outlined potential future studies that explore the broader applicability of this method. For more details, please refer to page 9 of the revised version of the manuscript.

Q3. Will the crystal crack affect the property in cascade-engineered approach?

Our reply: We thank the reviewer for pointing out this issue. We believe that cracks within the crystal significantly impact device performance. Surface cracks lead to larger surface defects, which can increase the carrier recombination rate, thereby reducing device efficiency. Additionally, if cracks manifest in the contact area, they can potentially cause device short-circuiting, severely compromising functionality. Therefore, fabricating high-quality single crystals is essential for minimizing these defects and ensuring

optimal device performance. This issue has been clarified in the revised version of the manuscript (see page 4).

Q4. The format of y axis in Figure 3a and Figure 3b is different from Figure 3c. Why does author separate the data in Figures 3a,b while merged data in Figure 3c?

Our reply: We thank the reviewer for the remark. We have updated the current unit on the y-axis in Figures 3a and 3b in the revised manuscript on page 8, as well as in Figure S4 in the revised supplementary information on page S5, to nanoamperes (nA). Please note that the IV curves for each device in Figures 3a and 3b are presented separately to clearly illustrate the changes in dark current for each cascade device. This format allows for easy observation of variations in the dark current signal and the quality of ohmic contact across the devices, enhancing the clarity and analytical depth of the results. Figure 3c consolidates the current-time (It) curves of the four devices to display the changes in dark current for cascade devices under a uniform bias voltage. This arrangement offers an intuitive comparison of dark current variations among different devices, effectively demonstrating the method's efficacy in managing dark current across cascade-connected devices.

Q5. Some recent reports on the X-ray detection and imaging can be given a short description to compare the present technology, and some references are shown herein, Light: Science & Applications, 2024, 13, 222.; Advanced Materials, 2023, 35, 2212022.; Advanced Functional Materials, 2023, 33, 5, 2210735.

Our reply: Thank you for your suggestion.We have incorporated additional references and expanded the descriptions as suggested. These updates can be found on page 2 of the revised manuscript.

Q6. Reference format should be re-checked; for instance, Adv. Func. Mater. should be Adv. Funct. Mater..

Our reply: Thank you for your correction. We have thoroughly reviewed and corrected the references throughout the article. The updates are included on pages 10-13 of the revised manuscript and on page S12 of the revised supplementary information.

Reponse to Reviewer 3

Comments

The results reported in this manuscript were interesting, but the novety of this mansucript was not well described.

Our reply: We thank the reviewer for the time and effort spent on reviewing our work**.** We would like to clarify and highlight that the central focus of this work is to introduce a novel strategy for X-ray device fabrication that addresses the issues of large dark currents and significant current drift observed in the most single crystals currently used in X-ray detectors, specifically $MAPbBr_3$ perovskite and 110-facet CdTe. This method stands out by being independent of the single crystal material, bias voltage, and thickness, offering low production costs and high potential for industrial application. Unlike the traditional dimensionality control, single crystal quality improvement, and heterojunction methods, this approach significantly enhances the detection limit through cascade connection of devices—an aspect not explored in existing literature—adding considerable practical value and novelty. To address this issue, we detail the advantages of this approach over traditional X-ray imaging technologies, particularly in improving image quality while reducing radiation exposure. This strategy holds significant promise for applications in medical diagnostics and low dose detection, potentially garnering widespread interest. In the revised manuscript, we emphasize the key concepts and innovations of our research, particularly on pages 3, 9, and 10, where we showcase the transformative potential of our approach.
